# The role of C:N:P stoichiometry in affecting denitrification in sediments from agricultural surface and tile-water wetlands

**DOI:** 10.1186/s40064-016-1820-6

**Published:** 2016-03-22

**Authors:** Brian D. Grebliunas, William L. Perry

**Affiliations:** Aaniiih Nakoda College, P.O. Box 159, Harlem, MT 59526 USA; School of Biological Sciences, Illinois State University, Normal, IL 61790-4120 USA

## Abstract

Nutrient stoichiometry within a wetland is affected by the surrounding land use, and may play a significant role in the removal of nitrate (NO_3_–N). Tile-drained, agricultural watersheds experience high seasonal inputs of NO_3_–N, but low phosphorus (PO_4_–P) and dissolved organic carbon (DOC) loads relative to surface water dominated systems. This difference may present stoichiometric conditions that limit denitrification within receiving waterways. We investigated how C:N:P ratios affected denitrification rates of sediments from tile-drained mitigation wetlands incubated for: 0, 5, 10, and 20 days. We then tested whether denitrification rates of sediments from surface-water and tile-drained wetlands responded differently to C:N ratios of 2:1 versus 4:1. Ratios of C:N:P (P < 0.05) and incubation length (P < 0.05) had a significant effect on denitrification in tile-drained wetland sediments. Carbon limitation of denitrification became evident at elevated NO_3_–N concentrations (20 mg L^−1^). Denitrification measured from tile water and surface water wetland sediments increased significantly (P < 0.05) at the 2:1 and 4:1 C:N treatments. The results from both experiments suggest wetland sediments provide a limiting pool of labile DOC to maintain prolonged NO_3_–N removal. Also, DOC limitation became more evident at elevated NO_3_–N concentrations (20 mg L^−1^). Irrespective of NO_3_–N concentrations, P did not limit denitrification rates. In addition to wetting period, residence time, and maintenance of anaerobic conditions, the availability of labile DOC is playing an important limiting role in sediment denitrification within mitigation wetlands.

## Background

Wetlands represent a useful mitigation tool to remove dissolved nutrients, particularly nitrate (NO_3_–N), in agricultural runoff from tiles or surface waters (Gale et al. 1993; Xue et al. 1998; Lund et al. 2000). However, the effectiveness of these wetlands varies widely, and wetland size relative to drainage area required to effectively reduce nitrate (NO_3_–N) in runoff ranges from 1:100 to 1:10 (Higgins et al. 1993; Woltemade 2000). This wide range of area described for effective NO_3_–N reduction may be at least in part due to the need for appropriate inputs of carbon, specifically dissolved organic carbon (DOC) and phosphorus (P) in addition to NO_3_–N. The availability of C and P can significantly affect the rates of denitrification (White and Reddy 1999; Dodds et al. 2004; Herrman et al. 2008), and in agricultural systems, where NO_3_–N concentrations are high, DOC and P may impart strong controls on denitrification rates. Thus, stoichiometric imbalances of C:N:P may limit denitrification in constructed wetlands and receiving streams.

Intensive row crop agriculture in the Upper Midwest has altered drainage patterns and influenced the concentration and stoichiometry of nutrient inputs to surface waters (Raymond et al. 2012). Many Midwestern agricultural fields, particularly in Illinois, have been drained with porous corrugated plastic and clay pipe that drains water from the field directly to the streams, bypassing many land surface mitigation structures (Lemke et al. 2011). Tile water can be directed from the streams to wetlands to reduce nitrate loading to streams, but this water has high NO_3_–N concentrations and low C and P concentrations (Royer and David 2005; Bernot et al. 2006; Vidon et al. 2008; Tank et al. 2010). In contrast, natural wetlands receive overland flow from the surrounding watershed and this water can maintain higher concentrations of DOC and lower NO_3_–N and P (Kaplan and Newbold 1993). The difference in nutrient stoichiometry is due to differential removal of DOC and P relative to nitrate as overland flow has shortened residence times relative to water slowly percolating through upper soil profiles like that of tile drained fields (Dahm et al. 1998). Therefore, surface water typically has elevated labile DOC due to limited reduction through soil and microbial processes (Chambers et al. 2010; Griffiths et al. 2009).

Phosphorus is the primary limiting nutrient in streams and lakes, and can constrain algal and microbial production at times (Vadstein et al. 2003). Labile DOC and P bind to fine particulate soils or are transformed through microbial processes limiting the input of each (Kalbitz and Kaiser 2007; Farahbakhshazad et al. 2008; USDA 2011). Denitrification is not limited by P when NO_3_–N is low, but when NO_3_–N exceeds 50 mg L^−1^, P additions have had significant positive effects (White and Reddy 1999). Nitrate concentrations (10–30 NO_3_–N mg L^−1^) are commonly higher than P concentrations (0.01–1.51 mg L^−1^) in many Midwestern watershed suggesting P limitation of microbial processes.

Carbon availability has been shown to affect denitrification in wetlands and is now being recognized as an important component of stream nitrogen cycling (Hume et al. 2002a, b; McCarty et al. 2007; Stelzer et al. 2014). Increased DOC inputs stimulate microbial growth and heterotrophic activity (Asmala et al. 2013). Seasonal flood pulses account for a large proportion of annual NO_3_–N delivery, along with elevated DOC concentrations, but the availability of DOC relative to NO_3_–N still limits denitrification (Sather 1992; Allison and Vitousek 2005; Reinhardt et al. 2006; Eimers et al. 2008). Sewage treatment plants that are required to remove NO_3_–N add DOC to maintain increased C:N ratios (>2:1) and commonly achieve nearly 100 % removal of NO_3_–N (Cherchi et al. 2009; Naik and Setty 2012). In agricultural systems, active watershed or wetland management may also be necessary to increase available carbon because of its potential to limit denitrification (Songliu et al. 2009). For example, woodchip bioreactors installed on tile outflows provide increased surface area and DOC for bacterial biofilms and can remove large amounts of NO_3_–N during low flows (Greenan et al. 2009; Jaynes et al. 2008). To optimize the efficiency of methods to reduce NO_3_–N concentrations, we need to better understand the extent to which NO_3_–N and P might limit biological nitrogen demand (BND) especially in systems with high NO_3_–N concentrations (>10 mg L^−1^ NO_3_–N).

The goal of this study was to examine the extent to which bacterial denitrification in wetland sediments was limited by NO_3_–N and P availability. We used replicated laboratory assays on wetland sediment from an established tile-drained wetland amended with assigned nutrient treatments to determine if DOC or P limited denitrification and to what extent. To test the potential role of DOC and P as limiting nutrients to denitrification, two NO_3_–N concentrations were tested (2 and 20 mg L^−1^), and DOC, P, or both were added to look for limiting and interactive effects. We hypothesized that increased DOC and P availability would increase bacterial denitrification potential. To further explore the role of DOC, we then examined bacterial denitrification rates in sediments from constructed, agricultural wetlands receiving surface water (elevated DOC) or tile water (low DOC) at low (2:1) and high (4:1) C:N amendments. We could than test whether the increased DOC inputs received by surface water wetlands reduces the extent to which denitrification is limited relative to tile drained wetland sediments. Due to their higher DOC concentrations, we also predicted that bacterial denitrification would be higher in surface water wetlands relative to tile water wetlands.

## Methods

### C:N:P study

To examine the response of microbial communities to altered C:N:P ratios we used homogenized sediments from a representative constructed wetland receiving tile water inflow. Sediments were collected from an 11-year old experimental constructed wetland complex within the Mackinaw River watershed near Lexington, IL (40° 38′ 23″ N, 88° 49′ 18″ W) in June 2011. Dissolved NO_3_–N concentrations in drain tiles commonly exceed 20 mg NO_3_–N L^−1^ in the spring, and inflow tile concentrations of dissolved reactive phosphorus (DRP) within our site ranged from <0.01 to 1.2 mg L^−1^. We removed the top layer of sediments (2 cm) from random locations within the wetland, homogenized the sediments into a single slurry and stored at 4 °C for 24 h. Sediment aliquots (20 g) were placed into individual 150 ml media bottles and sealed with a cap fitted with septa.

**Fig. 1:**
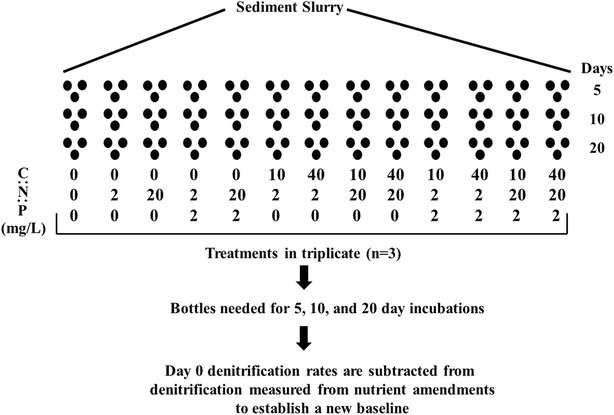
List of C:N:P amendments applied to drain tile wetland sediments and experimental outline

To test the effects of C:N:P, nutrient-amended water was added to microcosms and replaced daily by siphoning off overlying water. We used 16 nutrient ratios in triplicate that were destructively sampled for denitrification on days 0, 5, 10, and 20 (Fig. 1). Overlying water was amended with nutrient stock solutions of (0.1 M) potassium nitrate (KNO_3_), glucose-C (C_6_H_12_O_6_), and sodium phosphate (Na_2_HPO_4_). To provide favorable conditions for denitrifying bacteria, headspace of microcosms were flushed with N_2_ gas to remove oxygen and incubated at 25 °C on a 24-h dark cycle.

At the end of the nutrient incubation period, the acetylene inhibition technique was used to assess bacterial denitrification (Chan and Knowles 1979; Smith et al. 2006). Each replicate received 50 ml of ultrapure water and amended with 5 ml of a 0.1 M chloramphenicol solution. The headspace of each media bottle was than purged with N_2_ gas for 5 min to create anaerobic conditions. Acetylene (C_2_H_2_) gas, 15 ml, was injected into the headspace and shaken to incorporate into the soil. The addition of C_2_H_2_ blocks the conversion of NO_3_–N to N_2_ gas, therefore the end product for this assay is N_2_O. A 10 ml headspace gas samples was taken 15 min after the initial C_2_H_2_ injection, and once an hour for the following 4 h. Denitrification rates were calculated from the production of N_2_O h^−1^ g^−1^ of dried soil. Headspace gas samples collected throughout the assay were measured using a Shimadzu GC-2014 gas chromatograph with a Porapak Q packed column, detector temperature 300 °C, oven temperature 100 °C, and ultrapure nitrogen gas carrier was 10 ml min^−1^. Dry soil weight was obtained by drying the soil sample at 100 °C for 48 h after the incubation.

### Wetland type carbon study

**Fig. 2:**
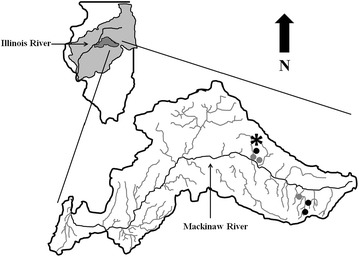
A map of the wetland sites used for sediment core collection within the Mackinaw River watershed, IL. The wetland denoted by an *asterisk* is the Gully wetland (40° 38′ 23″, 88° 49′ 18″) which was used for the C:N:P study. Sites incorporated into the wetland type study are denoted by circles on the watershed map as follows: surface water sites (*grey*) and tile water sites (*black*)

To test the response of microbial communities in surface versus tile-fed wetlands we conducted a similar microcosm study. We collected sediments from three wetlands receiving surface water (Frog, Floodplain East, and Floodplain West) and three wetlands receiving tile water (Moga, Gully, and Durbin) all of which were 6–7 years old (Fig. 2). All watersheds in this study were soybean and corn agricultural fields. Surface water wetlands receive water through extensive grass waterways and overland flow which was higher in DOC (2–200 mg L^−1^) relative to tile fed wetlands which were low (2–20 mg L^−1^) (McDowell et al. 1998; Royer et al. 2007).

To test for difference in bacterial denitrification between wetland sediments, we used two ratios of C:N, low (2:1) and high (4:1), and an unmanipulated control, each replicated 5 times. A C:N ratio greater than 4:1 was not tested due to wastewater studies finding it to be an effective ratio for high rates of denitrification (Sobieszuk and Szewczyk 2006). Nitrate–nitrogen concentrations in microcosms were maintained at 10 mg NO_3_–N L^−1^, which is commonly observed in tile water. DOC (glucose-C as C_6_H_12_O_6_) concentrations were 20 mg L^−1^ for the low C:N treatment and 40 mg L^−1^ for the high C:N treatment. Samples were destructively sampled on days 0, 5, 10, and 20 and assessed for denitrification as described above (Fig. 1).

### Data analysis

To examine differences in bacterial denitrification across each ratio of C:N:P tested, we used a two-way fixed effects analysis of variance with nutrient ratio and time as main effects. We used a mixed model ANOVA to test for differences in bacterial denitrification in the wetland type study with nutrient ratios, wetland type, and incubation time as the main effects and wetland sites as the random effect. Using Akaike Information Criterion (AIC_c_) we determined which parameters (or combination of multiple parameters) account for the most variation in denitrification rates (Burnham and Anderson 2002). When selecting the appropriate model, the parameters with the lowest AIC_c_ value are the best fit. Assumptions of normality and homogeneity of variances for both analyses were met without the aid of data transformations. The data analyses were performed using SAS 9.2 (SAS Institute Inc., 2008, Cary, North Carolina, USA).

## Results

### C:N:P study

**Fig. 3:**
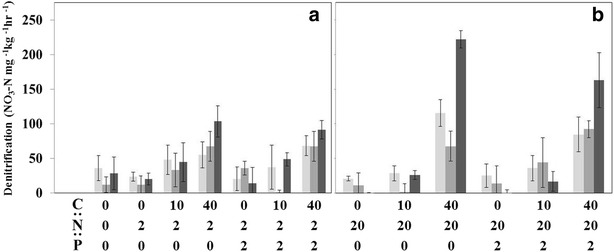
Response of denitrification to amendments of differing nutrient stoichiometry over time (5 days—*light gray*, 10 days—*medium gray*, and 20 days—*dark gray*). Denitrification rates measured on Day 0 were subtracted from subsequent days assayed to establish a new baseline. *Graphs* separated by low (**a** 2 mg L^−1^) and high (**b** 20 mg L^−1^) NO_3_–N availability

**Fig. 4:**
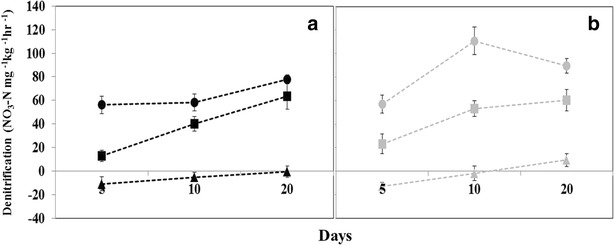
Shift in denitrification rates of tile (**a**) and surface water (**b**) sediments over time associated with different ratios of C:N. Denitrification rates from tile water sites are means of the Moga, Durbin, and Gully wetlands. Denitrification rates from the surface water sites are means of East Floodplain, Frog, and West Floodplain wetlands. Rates measured on Day 0 were subtracted from subsequent days assayed. Negative rates of denitrification represent a decrease in activity from Day 0. Treatments are denoted as follows: control (*filled triangel*), 2:1 (*filled square*), and 4:1 (*filled circle*). Wetland sediments from surface and tile water sites did not differ significantly in response to nutrient amendments

Bacterial denitrification differed significantly between nutrient ratios (F_15,143_ = 80.44, P < 0.0001). Denitrification was significantly greater in high C (40 mg L^−1^) and NO_3_–N (20 mg L^−1^), but denitrification did not differ when C was absent or low relative to NO_3_ (Figs. 3a, 4b). Denitrification rates also changed significantly over time (F_2,143_ = 20.29, P < 0.0001), however the directionality of the observed changes were dependent upon the microcosm stoichiometry (F_30,143_ = 5.75, P < 0.0001). Denitrification in elevated DOC (40 mg L^−1^) and NO_3_–N (20 mg L^−1^) increased significantly from days 0–20 (Fig. 3b). Denitrification in lower C:N ratios increased but the difference was not significant (Fig. 3b). In treatments without added C, denitrification rates decreased throughout the course of the study (Fig. 3a). Denitrification did not differ in treatments with added P (P > 0.05; Fig. 3a). With increasing NO_3_–N concentrations from the low to high treatment, denitrification rates decreased as the incubation progressed in the presence of P and not C (P > 0.05; Fig. 4). The low treatment (2 mg L^−1^) was stable and not significantly different over time, while the high treatment (20 mg L^−1^) decreased over time (P > 0.05; Fig. 3).

### Wetland type carbon study

The 2:1 and 4:1 C:N treatments resulted in denitrification rates significantly higher than that of the control (F_2,8_ = 148.09, P < 0.0001), and increases in denitrification rates did not differ between surface and tile water wetland sediments (F_1,4_ = 0.55, P = 0.5010; Fig. 4; Table 1). Denitrification rates increased rapidly in response to C:N treatments, but the increase was not significant over time (F_4,16_ = 1.9, P = 0.1592). Maximal rates of denitrification were reached earlier in the incubation when presented with the high C:N (4:1) treatment relative to the low C:N (2:1) treatment (Fig. 4). The C:N treatments resulted in similar maximal denitrification rates by day 20, although the 4:1 treatment achieved the highest rates by day 5 (Fig. 4). A significant interaction between sites, time, and treatment nested within wetland type (F_16,216_ = 2.11, P = 0.0090) suggested that denitrification differs over time dependent upon nutrient availability, and that wetland type appears to affect denitrification at elevated C:N. Denitrification also differed significantly between wetland sites within each wetland type (F_4,5.8_ = 6.42, P = 0.0246). The AICc supported the model that included the interaction of nutrients over time across wetland types and the primary factors affecting denitrification differs (Table 2).

**Table 1:** Results of the mixed model ANOVA showing there is no difference in denitrification between the high and low carbon treatments when tile and surface water wetlands are compared

Source	df	F	P
Wetland type error	1	0.55	0.5010

**Table 2:** Display of parameters tested, along with Akaike Information Criterion (AICc)

Model	AICc	Delta AICc	Exp function	wi
*TDS(W)*	*2373*	*0*	*1*	*0.8517*
DS(W)	2378	4.7	0.0953691	0.0812
TS(W), DS(W)	2379	5.1	0.0780816	0.0665
S(W)	2389	15.7	0.0000389	0.0003
TS(W)	2390	16.7	0.0002364	0.0002
Sum			1.1740769	

Values within the parameters including TDS(W) highlighted italics due to lowest AICc value, showing TDS(W) to be the best fit for the model. Model parameters labeled as follows: T (treatment), D (day), S (site), and W (wetland type)

## Discussion

### C:N:P study

The results of this laboratory study show that wetland sediments receiving elevated NO_3_–N levels commonly observed in central Illinois are strongly DOC but not P limited. Additions of NO_3_–N coupled with C appeared to alleviate the negative effects of NO_3_–N as the sole nutrient. Denitrification rates in control and low NO_3_–N treatments were stable over time, indicating that C from sediments was sufficient to meet bacterial demands. Denitrification in the presence of elevated NO_3_–N (20 mg L^−1^) decreased over the course of the experiment likely due to the limitation of DOC from sediments in high NO_3_–N. Upon the addition of elevated DOC coupled with NO_3_–N, denitrification increased significantly relative to all other treatments. These results highlight the need for added carbon in agricultural wetlands. Although DOC additions have been documented to lead to increased microbial activity, the extent to which bacteria were limited in agricultural watersheds, whether in streams or wetlands, can be better appreciated from a study manipulating the C:N:P ratios that are in the ranges observed in these systems (Hill and Cardaci 2004; Fork and Heffernan 2014).

Carbon limitation was evident in the presence of elevated NO_3_–N (20 mg L^−1^) concentrations, prompting potential management issues with the remediation of tile water in constructed wetlands. Under low NO_3_–N (2 mg L^−1^) and low DOC (10 mg L^−1^), denitrification rates remained stable over time (Fig. 3a). A shift from NO_3_–N limitation to DOC limitation was observed at the high DOC treatment (40 mg L^−1^) in the presence of low NO_3_–N resulted in an increase in denitrification by day 20, but the increase was minimal when compared to the elevated NO_3_–N treatment (20 mg L^−1^) (Fig. 3b). By design, constructed wetlands are installed to intercept a large catchment area relative to wetland size to maximize drainage retention, often resulting in extended periods of inundation (weeks to months) (Lee et al. 2002). Wetland areas that maintain pooled conditions exhibit elevated denitrification rates (and bacterial activity) relative to sediments that are temporarily inundate (Hernandez and Mitsch 2007). Coupling prolonged wetting periods with NO_3_–N saturation appears to have increased the demand for labile DOC throughout agricultural watersheds, likely reducing the effectiveness of wetlands as a mitigation tool due to energetic constraints.

Elevated rates of denitrification are often observed in wetlands following pulses of NO_3_–N inputs, but the associated spike in denitrification is more pronounced in low NO_3_–N (or N limited) systems (<5 mg L^−1^) (Forshay and Stanley 2005; Sirivedhin and Gray 2006; Smith et al. 2006). In high nitrate systems, elevated inputs of NO_3_–N often do not result in peaks in denitrification, which may be due to nitrate saturation (Mulholland et al. 2009). Seasonal pulses of water, often during the spring, offer elevated DOC for brief periods because of inputs from crop residue decomposition, but associated spikes in denitrification are often short relative to the input of NO_3_–N (Zarnetske et al. 2011). The allochthonous contributions of DOC are less prolonged than that of nitrate due to the limited pool of terrestrially derived carbon in agricultural landscapes (Guillemette and del Giorgioa 2011). Furthermore, autochthonous carbon is rapidly utilized and has minimal impact on NO_3_–N reductions in stream and wetland environments (Guillemette and del Giorgioa 2011). This would be particular evident in high NO_3_–N systems like the ones we have examined in central Illinois.

Tile inputs coupled with remobilization of P from anoxic wetland sediments appears to be sufficient for bacteria within the benthos of agricultural waterways since added P had no effect on denitrification. Aquatic systems limited by P can exhibit significant population growth of bacteria in response to low P concentrations (1–10 µg L^−1^) but growth asymptotes at the upper end of this range (Miettinen et al. 1997). Maximizing bacterial growth can play an important role in NO_3_–N reduction because a larger number of bacteria can translate to greater enzymatic production, for our purposes extracellular denitrifying enzymes. The lack of effect P had was likely due to the samples already being saturated with P due to the affinity of dissolved P to bind to organic materials in wetland sediments. Also, the availability of P does not provide energy to carry out the respiration of NO_3_–N, which appeared to be the primary limiting factor, rather than bacterial numbers (Correll et al. 1999).

Denitrification increased significantly in the presence of elevated C, while no effect of P availability was observed. Wetlands that receive elevated NO_3_–N inputs with little to no allochthonous DOC to supplement the autochthonous pool may experience reduced rates of denitrification due to DOC limitation (Burgoon 2001; Hume et al. 2002a, b). Establishing a matrix of emergent and submergent vegetation beds has been shown to increase denitrification, but C:N ratios of emergent macrophytes are similar to terrestrial plants leading to slow decomposition and minimal DOC inputs relative to NO_3_–N (Bachand and Horne 1999). Therefore, denitrification would likely increase if terrestrial contributions of labile carbon could be improved because the limited size of wetlands relative to watershed limit autochthonous DOC production.

### Wetland type study

Denitrification rates in sediments from surface and tile water wetlands did not differ significantly in their response to DOC additions (Fig. 4). The type of land use within a watershed serves an important regulatory role for the quantity and ratio of nutrients entering the receiving water bodies (Fraterrigo and Downing 2008; Abell et al. 2011). Surface waters entering wetlands from undisturbed uplands (prairie and forest) can have higher concentrations and more labile DOC relative to that of cropland runoff or tile drainage (Purakayastha et al. 2008). However, crop residues can contribute appreciable amounts of DOC during brief seasonal pulses of tile water (Royer and David 2005). We hypothesized that wetlands retaining surface water would exhibit substantially lower DOC limitation and higher denitrification overall. Our results suggest that denitrification rates did not differ significantly between wetland types in response to DOC additions. The uncharacteristically high inputs of NO_3_–N associated with row crop agriculture likely nullified the contribution of dissolved and particulate C fractions delivered in surface waters (Hussein et al. 2004; Inamdar et al. 2004).

We hypothesized that sediments from surface water wetlands might respond differently to added carbon and maintain elevated denitrification rates relative to tile-drained wetlands. However, denitrification in surface and tile water wetlands did not differ, and added DOC again significantly increased denitrification in all wetland sediments. These results underscore the importance of labile DOC for denitrification when NO_3_–N concentrations are high. These findings are consistent with the conclusions of more recent stream studies that have observed positive relationships between organic C availability and denitrification, and highlight how in high NO_3_–N systems this DOC limitation is more prevalent (Inwood et al. 2005, 2007).

The observed change in denitrification over time was contingent upon the C:N amendment, where the 4:1 treatment appeared to foster conditions that promoted a more rapid increase relative to the 2:1 treatment. This result suggests if brief pulses of row crop drainage had an elevated C:N, tile water may adequately enrich wetland sediments to meet the energetic demands of denitrifying bacteria. This would likely enable denitrification activity to more rapidly (≤5 days) respond to brief periods of NO_3_–N saturation (Fig. 4). In addition to requiring less time to reach maximum rates of denitrification, the 4:1 treatments maintained the elevated rates for the duration of the study, whereas the 2:1 treatment required 20 days to reach similar rates. The response to the low nutrient treatment is a more prolonged, linear increase and a similar trend was observed in both surface and tile water wetlands (Fig. 4). Rates of denitrification in each wetland type rapidly responded to pulses of DOC at high concentrations, suggesting the input of minimal allochthonous DOC has the potential to dramatically increase denitrification rates in high NO_3_–N systems.

The age of constructed wetlands may have some bearing denitrification rates. As wetlands age, the death and decomposition of aquatic plants and algae can lead to an accumulation of organic material over time (Mitsch et al. 2012). Low rates of denitrification due to limited DOC availability is particularly evident in newly constructed wetlands, but pulses of DOC in tile or surface water can lead to significant increases in denitrification (Song et al. 2011). The sites used for this study were 7–8 years old which is typically enough time to allow a sufficient amount of detrital accumulation and decomposition to meet heterotrophic carbon demands (Mustafa and Scholz 2011; Wolf et al. 2011). A low average DOC concentration coupled with a predominantly recalcitrant pool of DOC typical of mid-successional wetlands may be interacting to limit denitrification within agricultural wetlands. In landscapes with reduced inputs of NO_3_–N, constructed wetlands may be able to effectively reduce NO_3_–N to manageable concentrations. Further work is needed to investigate mechanisms to increase the availability of DOC within wetland environments, whether it comes from changes in agricultural practices or vegetation management within the wetlands themselves.

## Conclusion

Within intensely farmed watersheds, autochthonous and allochthonous DOC inputs associated with row crop agriculture are insufficient to support maximal denitrification when there is excessive NO_3_–N entering treatment wetlands. Without supplemental DOC, wetlands receiving prolonged periods of NO_3_–N laden tile water may reduce the NO_3_–N remediation potential. Denitrification rates had a distinct negative trend when sediments were presented with only NO_3_–N, which was most likely related to limiting heterotrophic conditions. This idea is further supported when rates of denitrification are improved with DOC additions as low as 2 mg L^−1^ over time. Irrespective of DOC or NO_3_–N concentrations, the addition of phosphorus did not have any significant interactive effects on denitrification rates. Although wetland sediments serve as phosphorus sinks, it was important to test for potential effects of phosphorus as it has been observed to limit denitrification in instances of extreme NO_3_–N contamination (White and Reddy 1999). However, inputs of DOC from surrounding terrestrial environments do not adequately support the energy needs of heterotrophs in the presence of NO_3_–N concentrations commonly observed within intensively farmed regions. Further work must be undertaken to address potential methods of increasing the amount of bioavailable C in order increase the effectiveness of wetlands as NO_3_–N removal tools, and potentially reducing the size of treatment wetlands making them a more feasible option throughout agricultural regions.
